# Understanding female sex workers’ acceptance of secret Facebook group for HIV prevention in Cameroon

**DOI:** 10.1371/journal.pdig.0000562

**Published:** 2024-08-14

**Authors:** Hassanatu B. Blake, Mercy Njah, Mary Mah Babey, Eveline Asongwe, Anna Junkins, Jodie A. Dionne, Ann E. Montgomery, Teneasha Washington, Nataliya Ivankova, Tamika Smith, Pauline E. Jolly

**Affiliations:** 1 Department of Health Behavior, School of Public Health, University of Alabama at Birmingham, Birmingham, Alabama, United States of America; 2 Cameroon Baptist Convention Health Services, Bamenda, Cameroon; 3 Department of Epidemiology, School of Public Health, University of Alabama at Birmingham, Birmingham, Alabama, United States of America; 4 Department of Infectious Diseases, School of Medicine, University of Alabama at Birmingham, Birmingham, Alabama, United States of America; 5 Department of Health Services Administration, School of Health Professions, University of Alabama at Birmingham, Birmingham, Alabama, United States of America; Iran University of Medical Sciences, ISLAMIC REPUBLIC OF IRAN

## Abstract

Despite the widespread utilization of social media in HIV prevention interventions, little is known about the acceptance of social media in the dissemination of HIV prevention information among key at-risk groups like female sex workers (FSWs). This study has investigated FSWs’ acceptance of Secret Facebook Group (SFG) in learning about HIV prevention. During June 2022, a quantitative study was conducted using a 5-star point Likert scale survey among 40 FSWs aged 18 years and older who took part in a Secret Facebook Group (SFG) HIV intervention. Descriptive statistics described demographics, social media accessibility, perceived usefulness (PU), perceived ease of use (PEOU), and acceptance among survey participants using SPSS and SAS. Most study participants found SFG utilized in HIV prevention intervention acceptable. Seventy-five percent (75%) of participants selected 5 stars for the acceptance of SFG. The majority of participants used social media, spent more than 90 minutes on social media per day, and could participate in the SFG HIV prevention intervention if airtime was not provided by study investigators, despite experiencing times when the internet was interrupted. The results also showed the PU and PEOU mean scores of SFG in the HIV prevention intervention were slightly lower than the acceptance scores (4.70 and 4.50 vs. 4.74). The data suggested future research should focus on explaining FSWs acceptance of social media and identifying social media platform alternatives for HIV prevention intervention. This study provided useful insights into social media acceptance, use, and importance in HIV prevention education among FSWs. The findings also indicate the need for further research on the reasons for acceptance of social media and relevant social media platforms supporting HIV prevention education among FSWs.

## Author summary

Globally, HIV prevention efforts have faced challenges in reaching marginalized communities, who often lack access to HIV education and care services. Digital health solutions present opportunities to address this gap and provide a discreet channel for fostering peer support and sharing HIV education and resources. Our study focused on understanding the acceptance and usability of a Secret Facebook Group intervention by Cameroonian female sex workers in learning about HIV prevention. Our findings revealed a positive response to the Secret Facebook Group intervention, with 75% of participants rating the acceptance of the Secret Facebook Group intervention at the highest level of 5 stars out of a 5 stars scale. Most participants were familiar with social media and spent substantial time online daily, despite internet disruptions. In addition, the high ratings of perceived usefulness and ease of use of Secret Facebook Group highlight the potential of social media as a valuable tool in HIV prevention education. Future research should study the nuances of social media acceptance in HIV prevention education and explore alternative platforms that may better resonate with female sex workers.

## Introduction

In many sub-Saharan countries, including Cameroon, FSWs have limited access to sexual and reproductive health interventions including HIV prevention education because sex work is illegal in those countries [1]. The criminalization of sex work hinders efforts to prevent HIV and other STIs among FSWs [1]. Globally, the use and awareness of pre-exposure prophylaxis (PrEP) have been proven effective in HIV prevention among FSWs [2]. Individual-level interventions such as personal messages and behavior change tools can be modified for delivery via mobile devices.

Mobile technologies are found to be cost-effective [3], and have the potential to combat barriers to access prevention tools and improve outcomes [4]. In a 2016 study by the Global Network of Sex Work Projects, sex workers from 7 low- and middle-income countries (LMICs) and high-income countries shared that the predominant method for accessing ICTs was found to be through a smartphone [5]. The use of smartphones to access ICT enhanced social cohesion among sex workers [5]. Sex workers in the United Kingdom used the text messaging app WhatsApp (Facebook, Inc) to form private chat groups to exchange information on clients [5].

In addition to WhatsApp, Secret Facebook Group (SFG) is a Facebook group that is not visible to anyone through Facebook searches or the outside world. Participants join the group only by an invitation from a Facebook friend who is also a member of the group. Increased social support through this kind of social media platform has improved access to HIV prevention services and builds a community with peers, which is a critical component in controlling the spread of HIV [6]. Two quantitative studies conducted in China examined the feasibility of using the internet to communicate HIV prevention information to indoor cisgender female sex workers [5]. In one of the studies, a small percentage of sex workers (6.7%) reported consistent condom use after seeing public service announcements [5]. In the other study, many sex workers surveyed (64%) shared that they would join a web-based HIV and sexually transmitted infection prevention program [5]. Despite the widespread utilization of social media in HIV prevention interventions, very little is known about the acceptance of social media in the dissemination of HIV prevention information among key at-risk groups like FSWs.

Acceptance reflects the thoughts and feelings of participants about the intervention’s technology *after* engagement [7,8,9]. Acceptance is strongly related to beliefs about the effects of the technology, service, and tools after implementation or introduction of the intervention to a group [10]. To measure the influence of factors and construct a comprehensive model for acceptance of social media, the Technology Acceptance Model (TAM) was combined with the Theory of Planned Behavior (TPB) to build the theoretical foundation for this research [11].

Many studies use the combined Technology Acceptance Model and Theory of Planned Behavior (C-TAM-TPB) to explain the adoption of technology, and this has proven useful in studies regarding the acceptance of digital health interventions [12]. The C-TAM-TPB postulates perceived usefulness (PU) and perceived ease of use (PEOU) to have direct and positive effects on behavioral intention as a valid predictor of acceptance [13].

During the COVID-19 pandemic, there was an increased use of digital health modalities, particularly social media [14]. Therefore, it is timely to explore acceptance of social media in areas of health intervention. Earlier studies show that acceptance of technology influences behavior and is affected by a variety of factors including individual differences, social and situational influences, user beliefs, user attitudes, and managerial intervention [15]. A 2021 randomized pilot project evaluated an intervention that complemented existing HIV prevention services among HIV-negative FSWs in Cameroon [16]. The intervention was a HIV and sexual health video curriculum consisting of 12 videos promoting health care literacy around HIV, pre-exposure prophylaxis (PrEP), post-exposure pro phylaxis (PEP), condom use, sex work information from around the globe, and stigma reduction tailored to FSWs [16]. Each video was no longer than 2 minutes in duration and all videos were released over 8 weeks through a secret Facebook group platform and surveys were administered before, after the intervention, and three months later [16]. A finding of this project is that all participants recommend the intervention to fellow FSWs [16].

Despite this intervention, only limited research on the use of social media in HIV prevention education among FSWs has been conducted. By leveraging the findings of the aforementioned project, this study aimed to evaluate the utility and acceptance of Secret Facebook Group (SFG) in learning about HIV prevention by FSWs in Cameroon.

## Methods

### Study design and participants

During June 2022, 40 participants were recruited via phone and in-person at New Life Club, an organization providing resources to sex workers in Cameroon. The eligibility criteria for participation in the study were that FSWs had to be of adult age (18 years or older), be a member of NLC, participated in the SFG HIV prevention education intervention in 2021, had good command of the English language, and possessed a smartphone.

Secret Facebook Group (SFG) was chosen as the study vehicle for the SFG HIV prevention intervention in 2021 because it was a free social media platform that protected participant privacy [16]. Participants’ privacy was a priority given that sex work is an illegal activity in Cameroon [16]. Therefore, the more commonly used WhatsApp platform was not used as it requires personal identification, such as phone numbers, to be shared [16]. Though all participants of the study knew other members were FSWs, all participants adopted pseudonyms on the online Facebook account used to join the SFG and a study number for this study. Content in Secret Facebook Group (SFG) is visible only to SFG members, but Facebook/Meta does reserve the right to access content under certain policy violations or legal circumstances. The study participants shared content and communicated only within the SFG. No participant of the group was allowed to screenshot, reshare, or download content from SFG to protect user’s privacy and data. The study team also served as administrator and moderators of the SFG to monitor SFG rules and adherence.

NLC Coordinators told all participants about the length of time of the survey and all participants gave written informed consent before they completed the survey, which took approximately 30 minutes. Participation was voluntary and participants could withdraw from the study at any time. Financial assistance was provided for smartphone airtime and roundtrip transportation to and from NLC. No personal information was collected or stored.

The study team developed the paper survey and NLC coordinators administered it to the participants. Prior to survey administration, NLC coordinators completed training related to interview skills, audiotaping, and storing data via Zoom and WhatsApp. The training sessions also incorporated a practice session with the first author to accurately convey the same meaning for each survey question to ensure fidelity in administration of the interviews during the practice session. The study was approved by the Cameroon Baptist Convention Health Services Institutional Review Board (CBCHS IRB) and the University of Alabama at Birmingham Institutional Review Board.

### Measures

The star rating questionnaire contained the following items: sociodemographic factors (i.e., age, education, date of birth, marital status, and children/offspring); health information (i.e., HIV status since the parent study and an assessment of awareness of HIV status, PrEP awareness); accessibility of social media (i.e., social media history and preferences); and acceptance of social media in HIV prevention (perceived usefulness, ease of usage, acceptance).

The star ratings survey can capture reactions to digital health interventions and factors that influence them [17]. Sociodemographic data were assessed using items on age, marital status, having children (aged < 18 years), educational level, and occupational status. Regarding HIV status, participants were asked whether they knew their status, what their status is, had they ever been tested for HIV, had they ever been told they have HIV, and were they aware of PrEP before the SFG HIV prevention education intervention. In terms of accessibility of social media, participants were asked about their ownership of smartphones, time spent on a smartphone, access to the internet via smartphone, social media usage, and family and friends’ social media usage.

In addition, the questionnaire content was based on the Mobile Application Rating Scale (MARS), and the Technology Acceptance Model (STAM) Scale. MARS is a widely used tool to evaluate the quality of mobile health apps. STAM Scale is a usability scale to assess behavioral intention to use an intervention’s technology. The survey’s 5-star scale ranged from “1 star for Strongly Disagree” to “5 stars for “Strongly

Agree”. Study participants provided their ratings of SFG by selecting a number of stars ranging from 1 to 5 for each question or statement. Each research measure (perceived usefulness, ease of usage, and acceptance) had associated questions or statements.

The Perceived Usefulness (PU) variable included 16 survey statements and the Perceived Ease of Usage (PEOU) variable had 10 survey statements. Acceptance was defined as behavioral intention to use SFG in HIV prevention education and included five associated survey statements and questions. A mean score was calculated for each variable category. The overall SFG acceptance score was the mean of perceived usefulness, ease of usage, and acceptance indicators. An overall score of 4 stars or above was considered acceptance of SFG in the parent project. Further details on Constructs and Research Questions to the survey can be found in S1 Appendix.

### Descriptive Statistics

Quantitative data from 40 survey respondents were entered and imported into SPSS 26 (IBM) and SAS for analysis. Descriptive statistics were calculated for demographic characteristics of participants using mean ± standard deviation for continuous variables and frequency (percentage) for categorical variables.

Next, acceptance was computed, its distribution assessed, and respective frequencies calculated.

## Results

### Demographics

Forty participants, aged 22 to 56 years, completed the survey (Table 1). On average, participants were 30.65±SD years; most were single (95%), had a secondary education or higher (70%), and had children (77.5%). The average number of years the women had been working as sex workers was 9.43±SD and 47.5% of the women who worked as full-time FSWs.

**Table 1:** Demographic Characteristics of Study Participants (N = 40).

Variable	Number (%)
**Age**	
21 years or less	4 (10.0)
22–29 years	19 (47.5)
30 years or more	17 (42.5)
**Years working as CSW**	
2–5 years	5 (12.5)
More than 5 years	35 (87.5)
**Type of Work**	
No response	8 (20.0)
Part time	13 (32.5)
Full time	19 (47.5)
**Number of Children**	
None	9 (22.5)
1	8 (20.0)
2	7 (17.5)
3 or more	16 (40.0)
**Number of living children**	
None	9 (22.5)
1	8 (20.0)
2	6 (15.0)
3 or more	17 (42.5)
**Number of children living with participant**	
None	8 (20.0)
1	3 (7.5)
2	4 (10.0)
3 or more	25 (62.5)
**Number of pregnancies**	
None	8 (20.0)
1	4 (10.0)
2	8 (20.0)
3 or more	20 (50.0)
**Marital Status**	
Single/Widowed	39 (97.5)
Married	1 (2.5)
**Education level**	
Primary	12 (30.0)
Secondary	20 (50.0)
More than Secondary	8 (20.0)

CSW, Commercial Sex Worker

### HIV status

HIV status and prevention awareness are displayed in Table 2. All participants self-reported that they had been tested for HIV, knew their HIV status, and were HIV negative. Most women were not aware of PrEP before the SFG HIV prevention education intervention (87.2%).

**Table 2:** HIV Status and PrEP Awareness (N = 40).

HIV Status & PrEP Awareness	N (%)
Tested for HIV	40 (100.0)
Knew their HIV Status	40 (100.0)
HIV negative	40 (100.0)
Not told previously that they had HIV	38 (95.0)
Not aware of PrEP before SFG HIV prevention education intervention	35 (87.2)

HIV, human immunodeficiency virus; PrEP, pre-exposure prophylaxis; SFG, Secret Facebook Group

### Accessibility of Social Media

Accessibility of social media variables were also measured (Table 3). All FSWs had access to the internet via smartphone, used social media, had access to social media via their smartphone, used Facebook, felt comfortable conducting social media activities, and had family and friends who used social media. The majority of participants (82.5%) did not share a smartphone with anyone, spent three or more hours per day using the internet via their smartphone (55%), experienced times of internet interruptions (70%), and bought airtime to use internet two or more times per week (60%). The majority of FSWs would participate in the SFG HIV prevention intervention if airtime was not provided (55%).

**Table 3:** Accessibility and Use of Social Media (N = 40).

Accessibility & Use of Social Media	N(%)
Use internet via smartphone	40 (100.0)
Buy airtime to use internet	40 (100.0)
Would participate in online intervention if airtime was not provided	40 (100.0)
Length of time on social media per day 90 min or more	29 (72.5)
Owned a smartphone	40 (100.0)
Access to the internet via smartphone	40 (100.0)
Used social media	40 (100.0)
Access social media via their smartphone	40 (100.0)
Used Facebook	40 (100.0)
Comfortable conducting social media activities	40 (100.0)
Family and friends use social media	40 (100.0)
Did not share a smartphone	33 (82.5)
Use the internet via their smartphone for 3 more hours per day	22 (55.0)
Internet interruptions	28 (70.0)
Bought airtime to use internet two or more times per week	24 (60.0)
Participate in the SFG HIV prevention intervention if airtime was not provided	22 (55.0)
Use WhatsApp	35 (87.5)
Spent more than 90 mins on social media per day	29 (72.5)
Watched videos on social media	39 (97.5)
Text or message with friends or clients	28 (70.0)
Safe conducting social media activities	39 (97.5)
Believed personal information is kept private	39 (97.5)
Preferred the social media apps Facebook WhatsApp	20 (50.0) 19 (47.5)
Immediate family who preferred to use Facebook	33 (82.5)
Friends who preferred to use Facebook	36 (90.0)

HIV, human immunodeficiency virus; SFG, Secret Facebook Group

In addition, most FSWs reported they currently used social media (97.5%), used WhatsApp (87.5%), spent more than 90 minutes on social media per day (72.5%), watched videos on social media (97.5%), and texted or messaged with friends or clients (70%). Participants expressed feeling safe conducting social media activities (97.5%), believed that personal information was kept private (97.5%), preferred the social media apps Facebook (50%) and WhatsApp (47.5%), had immediate family members who preferred to use Facebook (82.5%), and had friends who preferred to use Facebook (90%).

### PU Score

Table 4 displays FSWs’ perceived usefulness (PU) of SFG in HIV prevention education. The mean PU score was 4.70 (SD = .49). Forty percent (40%) of participants reported a score of 5 for the PU of SFG.

**Table 4:** Outcomes of SFG Acceptance.

Outcomes	Mean (SD)
Perceived usefulness (PU)	4.7 (0.5)
Perceived Ease of Use (PEOU)	4.5 (0.6)
Acceptance	4.7 (0.5)

SD, standard deviation; SFG, Secret Facebook Group

### PEOU Score

The mean PEOU score was 4.50 (SD = .59). Twenty percent (20%) of participants reported a score of 5 for the PEOU of SFG.

### Acceptance Score

Three-quarters (75%) of participants reported an acceptance score of 5. The mean acceptance score was 4.74 (SD = .53).

## Discussion

Results from the descriptive analysis showed the mean scores for FSWs’ perceived usefulness, perceived ease of use, and acceptance of SFG in the HIV prevention intervention as 4.70, 4.50, and 4.74, respectively. The survey revealed that most participants used social media, spent more than 90 minutes on social media per day, and could participate in the SFG HIV prevention intervention if airtime was not provided, despite experiencing times when the internet was interrupted.

Poor connectivity and internet interruption were common in Cameroon, which negatively impacted access and use of social media. There has been an increasing internet penetration rate in Cameroon [18]. Though many in Cameroon use mobile phones, internet penetration rate in Cameroon was calculated to be 34% in 2021 and accessed mainly in urban areas (75% of users) [19]. Due to the low internet penetration in Cameroon, participants of this study who were located in urban areas of Cameroon still experienced poor connectivity and internet interruption. Participants and their family and friends preferred Facebook and WhatsApp to other social media applications because they felt safe sharing information and interacting with one another using readily available and free applications. Studies conducted to understand the perceived benefits of social media use reported that sex workers are safer and stronger because of social media [20]. A cluster randomized controlled trial study with two arms—one arm receiving the text messaging intervention and the other receiving verbal HIV prevention information at the clinic—was conducted in South Africa, Zimbabwe, and Mozambique. Though the study did not use SFG, it did use text messaging that led to an increase in self-reported HIV testing among sex workers in the intervention group [5]. With social media, FSWs can connect with their peers, family, and friends without explicitly identifying as FSWs [21]. Not only do the initial data reveal the acceptance of SFG, but they also provide a better understanding of the social media acceptance in HIV prevention education. The PU and PEOU mean scores of SFG in the HIV prevention intervention were slightly lower than the acceptance score (4.70 and 4.50 vs. 4.74).

The perceptions of usefulness and ease of use of SFG did not negatively influence FSWs’ perceptions of acceptance of SFG. Several factors could contribute to this situation. The majority of women had three or more living children and children who reside with them, which may have influenced their acceptance of SFG. Since the youth population is the largest group to use social media, they could influence their parents’ or guardians’ acceptance of social media. In addition, prior awareness, and use of social media by participants may have led them to rate acceptance of SFG high, despite its perceived usefulness and ease of use.

The slightly lower PU and PEOU may be attributed to unstable internet connectivity and lack of infrastructure in Northwest and Southwest Cameroon. These data also identify areas for future research to inform design of social media-based HIV prevention education interventions. Specifically, future work should focus on the reasons for FSWs acceptance of social media and social media platform alternatives for HIV prevention intervention.

### Limitations

This study has limitations that must be considered in interpreting the results. First, the sample size was only 40 participants and surveys had a couple of missing responses to some variables. With such a small sample, additional statistical analyses could not be performed. Second, the study sample represented only FSWs who were recruited by NLC and participated in SFG HIV prevention intervention conducted in Western Cameroon a year prior to this survey. Therefore, the results may not be generalizable to FSWs who did not participate in the SFG HIV prevention intervention and FSWs in other areas of Cameroon.

Additionally, the data were self-reported and may be subject to social desirability bias.

## Conclusion

This study showed that Secret Facebook Group was an acceptable tool in HIV prevention education for FSWs who participated in SFG-based HIV prevention education intervention. The findings from the survey provided the latest information and insights into the factors, such as perceived usefulness and perceived ease of use, influencing the behavioral intention of participants to use social media. The FSWs rated SFG’s usefulness, ease of use, and acceptance highly. These findings highlight the importance of social media HIV prevention education interventions among FSWs and other key populations.

Many participants had the ability to buy airtime that allowed them to share information via Facebook and WhatsApp with their family, friends, and clients. Though this sample size was very small, this study provided new information on the perceived usefulness, perceived ease of use and acceptability of SFG for HIV prevention. The results of a qualitative study conducted to provide research findings on social media-based HIV prevention interventions targeting FSWs will be reported. In addition, findings can be used to inform, develop, and improve appropriate interventions using relevant social media platforms to support HIV prevention learning among FSWs.

## Supporting information

S1 Fig Adapted C-TAM-TPB Model. Conceptual framework used to guide this study and related scoping review and qualitative study.
(TIF)

S2 Fig VIDEO LESSON SAMPLE TRANSCRIPT. An example of one of the modules shared with participants in the SFG intervention.
(TIF)

S1 Appendix Constructs and Research Questions. Questions based on the adapted C-TAM-TPB Model.
(DOCX)
