# Supplementary figures and images for: Understanding female sex workers’ acceptance of secret Facebook group for HIV prevention in Cameroon
Source: PLOS Digit Health. 2024 Aug 14;3(8):e0000562. doi: 10.1371/journal.pdig.0000562 (PMC11324143; doi:10.1371/journal.pdig.0000562)

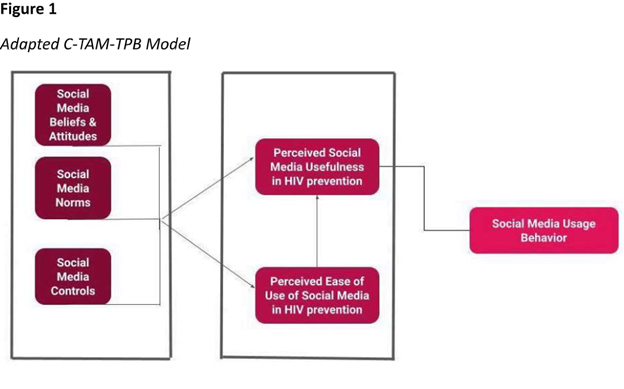

Supplement: S1 Fig — Conceptual framework used to guide this study and related scoping review and qualitative study. (TIF) [file pdig.0000562.s001.tif]

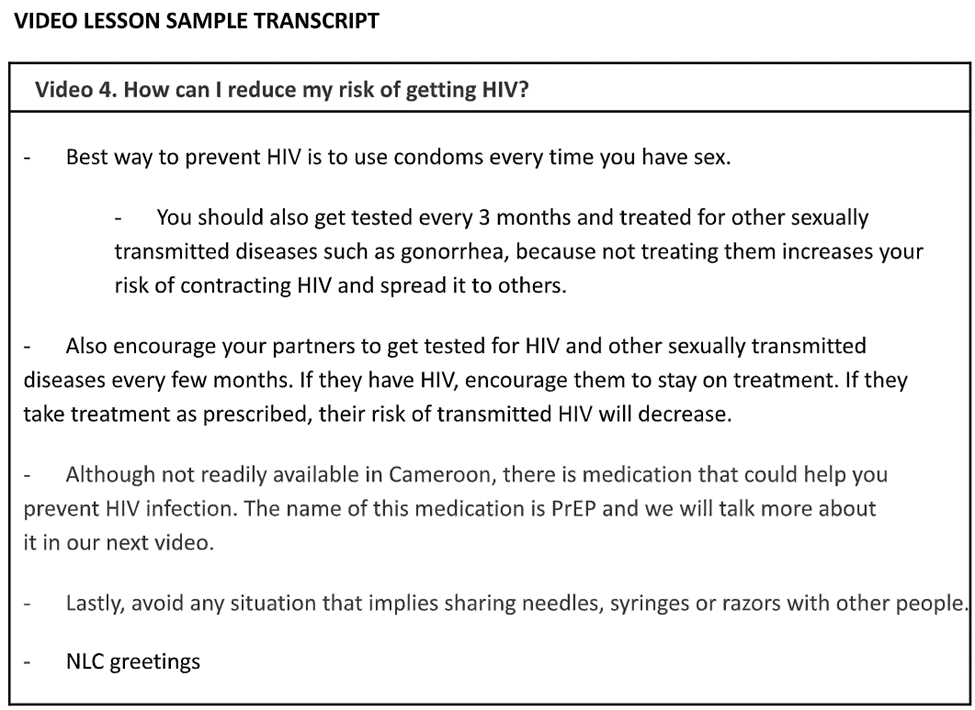

Supplement: S2 Fig — An example of one of the modules shared with participants in the SFG intervention. (TIF) [file pdig.0000562.s002.tif]
