# Supplementary material for: Understanding female sex workers’ acceptance of secret Facebook group for HIV prevention in Cameroon
Source: PLOS Digit Health. 2024 Aug 14;3(8):e0000562. doi: 10.1371/journal.pdig.0000562 (PMC11324143; doi:10.1371/journal.pdig.0000562)
Supplement: S1 Appendix — Questions based on the adapted C-TAM-TPB Model. (DOCX) [file pdig.0000562.s003.docx]

**APPENDIX**

**Constructs and Research Questions.**

| **Construct** | **Research Questions** |
| --- | --- |
| Behavioral intention  (Acceptance) | **Star Rating Questions**  I believe the use of SFG in the NLC Project was a good idea.  I was satisfied with the SFG used in the NLC Project.  I will use SFG more to learn about HIV prevention strategies.  I would recommend SFG to others who want to learn about HIV prevention strategies.  What is your overall star rating of SFG? |
| Perceived usefulness  (PU) | **Star Rating Questions**  I find social media helpful to learn about HIV prevention and PrEP.  I find SFG helpful during the NLC Project.  I find it helpful to receive reminders of HIV prevention video releases in the SFG.  I find social media helpful for me to educate others about HIV prevention.  I find SFG useful in my HIV prevention activities.  I am satisfied with HIV prevention education from SFG.  I find my use of SFG in the NLC Project has increased my awareness and knowledge of PrEP and HIV prevention.  I find using SFG would enhance my effectiveness in HIV prevention activities. |
|  | I find my use of SFG in the NLC Project has changed my attitudes toward improving practices against HIV.  I find my use of SFG in the NLC Project has increased my intentions/motivation to practicing HIV preventive measures.  I find my use of SFG in the NLC Project is likely to encourage me to further seek help for HIV testing, care, and treatment. |
| Perceived ease of use  (PEOU) | **Star Rating Questions**  I like the idea of using SFG.  I could be skillful at using SFG.  I find when I want or need to use SFG, it is accessible.  I feel apprehensive about using SFG.  I hesitate to use the SFG for fear of making mistakes I cannot correct.  I find SFG interesting to use.  I find that SFG allows user input, feedback, and prompts (reminders, sharing options, notifications, etc.)?  I find SFG features (functions) and components (buttons/menus) to work fast.  I find it easy to learn how to use SFG.  I find SFG menu labels/icons and instructions clear.  I would recommend SFG to others who want to learn about HIV prevention strategies.  What is your overall star rating of SFG? |
